# Paeonol attenuated high glucose-induced apoptosis via up-regulating miR-223-3p in mouse cardiac microvascular endothelial cells

**DOI:** 10.1038/s41598-024-67721-3

**Published:** 2024-07-19

**Authors:** Bo Deng, Ruyu Xian, Yuan Shu, Haohan Xia, Chengcheng Feng

**Affiliations:** 1 Department of Endocrinology, The Third Affiliated Hospital of Nanchang University, Nanchang, Jiangxi China; 2 School of Medicine, Nanchang University, Nanchang, Jiangxi China

**Keywords:** Paeonol, High glucose, miR-223-3p, Endothelial cells, Cell biology, Drug discovery, Diseases, Endocrinology

## Abstract

To investigate the role of miR-223-3p in the modulatory effect of paeonol (Pae) on high glucose (HG)-induced endothelial cell apoptosis. HG (25 mmol/L) was used to induce cellular damage and apoptosis in the mouse cardiac microvascular endothelial cells (MCMECs). Various concentration of Pae was tested and 60 μmol/L Pae was selected for the subsequent studies. MCMECs were transfected with exogenous miR-223-3p mimics or anti-miR-223-3p inhibitors. Cell viability was assessed by MTT assay and apoptosis was quantified by flow cytometry. The expression of miR-223-3p and NLRP3 mRNA was measured using real-time quantitative RT-PCR, and protein level of NLRP3 and apoptosis-related proteins was detected by immunoblotting. Pae significantly attenuated HG-induced apoptosis of MCMECs in a concentration-dependent manner. In addition, Pae (60 µmol/L) significantly reversed HG-induced down-regulation of miR-223-3p and up-regulation of NLRP3. Pae (60 µmol/L) also significantly blocked HG-induced up-regulation of Bax and Caspase-3 as well as down-regulation of Bcl-2. Moreover, exogenous miR-223-3p mimics not only significantly attenuated HG-induced apoptosis, but also significantly suppressed NRLP-3 and pro-apoptotic proteins in the MCMECs. In contrast, transfection of exogenous miR-223-3p inhibitors into the MCMECs resulted in not only significantly increased apoptosis of the cells, but also significant suppression of NLRP3 and pro-apoptotic proteins in the cells. Pae attenuated HG-induced apoptosis of MCMECs in a concentration-dependent manner. MiR-223-3p may mediate the modulatory effects of Pae on MCMEC survival or apoptosis through targeting NLRP3 and regulating apoptosis-associated proteins.

## Introduction

Atherosclerosis (AS) is a disease that affects arterial wall causing thickening or functional degeneration, and is characterized by endothelial cell injury, lipid metabolism disorder and inflammatory cell infiltration^1^. It is also the pathological basis of various cardiovascular diseases^2,3^. Dyslipidemia and smoking, hypertension, diabetes, and obesity have been traditionally considered to be risk factors for AS, which cause vascular endothelial injury and abnormal function^4^. Vascular endothelial injury is the initial stage of AS, which triggers the expression of several inflammatory factors and adhesion molecules that promote its further development^5^.

MicroRNAs (miRNAs) are small-molecule non-coding RNAs involved in regulating gene expression post-transcriptionally and various biological processes such as cell differentiation, proliferation, and metabolism^6^. Until recently, miRNAs were believed to regulate the expression of their target mRNAs only in the cells that produce them. It is now known that miRNAs can also be secreted into the circulatory system to affect various target organs throughout the body^7^. Thus, miRNAs in serum or body fluids are potential biomarkers for early diagnosis of various metabolic diseases and potential therapeutic targets in obesity, diabetes, and atherosclerosis treatment^8^. We have previously reported that overexpression of miR-223-3p could reduce injury in MCMECs and suppress endothelial cell apoptosis in mice^9^.

Paeonal (Pae), the main active ingredient extracted and purified from moutan, a Chinese herbal medicine, exhibits broad pharmacological activities including antioxidant, anti-apoptotic, anti-inflammatory, and analgesic properties^8,10,11^. Therefore, the current study was designed to investigate the effect of Pae on endothelial cell survival or apoptosis in the presence of HG, as well as the potential role of miR-223-3p in mediating the modulatory effect of Pae on HG-induced endothelial survival or apoptosis.

## Materials and methods

### Materials

Paeonol (98% purity) was obtained from Yuanye Bio-Technology Co., Ltd (Shanghai, China). Mouse cardiac microvascular endothelial cells (MCMECs) were purchased from Wuhan Procell Life Science & Technology Co., Ltd (Wuhan, China). The DMEM medium was purchased from HyClone (Logan, UT, USA), and the fetal bovine serum (FBS) and trypsin–EDTA were purchased from Sigma (St. Louise, MO, USA). Trizol and Lipofectamine™2000 were purchased from Invitrogen (Waltham, MA, USA). MTT assay and reverse transcription PCR test kits were purchased from Boster Biological Technology Co., Ltd (Wuhan, China) and TaKaRa (Kusatsu, Shiga, Japan), respectively. Antibodies against NLRP3, Bax, Caspase-3 and Bcl-2 were purchased from Abcam (Shanghai, China). Annexin V-FITC/PI apoptosis detection kit was purchased from Becton Dickinson and Company (Franklin Lakes, NJ, USA). RT-qPCR primers were purchased from JRDUN Biotechnology Co., Ltd (Shanghai, China). MiR-NC, miR-223-3p mimics, anti-miR-223-3p and anti-miR-NC were purchased from Sangon Biotech Co, Ltd (Shanghai, China).

### Methods

#### Cell culture and treatment

Cells were grown in the DMEM medium supplemented with 10% FBS and Penicillin/Streptomycin. After achieving 80–90% confluence, cells were detached with 0.25% trypsin containing EDTA. The cell suspension was then inoculated into the flask in a ratio of 1:2 split and incubated at 37 °C with 5% CO_2_. Cells in logarithmic growing phase were inoculated into 6-well plates and treated as followings: (1). Control group: 5.5 mmol/L glucose in the culture medium. (2). High glucose (HG) group: 25 mmol/L glucose in the culture medium. (3). HG + Pae group: cells in this group were initially treated with three different concentrations of Pae (30, 60, and 120 µmol/L, respectively) in addition to HG (25 mmol/L). Afterwards, 60 µmol/L Pae was used for the subsequent experiments.

#### Transfection of miRNA mimics and inhibitors

Using lipofectamine™2000 transfection reagent, the cells were transfected with either negative control of miRNA mimics (miR-NC), mimics of miR-223-3p (miR-223-3p), negative control of miRNA inhibitor (anti-miR-NC), or specific inhibitor of miR-223-3p (anti-miR-223-3p). Expression of miR-223-3p was examined 48 h after the transfection. Cells were then treated with high glucose (HG) with or without Pae (60 µmol/L).

#### MTT assay

After the treatment of the cells with HG with or without Pae, a final concentration of 1 mg/ml MTT solution was added to each well and incubated for 4 h followed by the addition of 100 μL dimethyl sulfoxide to dissolve the crystals. Viability of the MCMECs was evaluated based on absorbance acquired at 570 nm using a scanning multi-well spectrophotometer.

#### Apoptosis analysis

Apoptosis of the MCMECs was measured using flow cytometry analysis. Briefly, after digestion, the cells were centrifuged at 1000 r/min for 5 min and washed twice in pre-cooled PBS. The cells were re-suspended in 1 × binding buffer and mixed with 2.5 µl Annexin V-FITC and PI followed by incubation in the dark at room temperature for 15 min. Data were analyzed using FlowJo software.

#### RNA extraction and qRT-PCR assay

Total RNA of MCMECs was extracted using the Trizol method and then reverse transcribed into cDNA followed by amplification by PCR using β-actin and U6 as the internal reference control for NLRP3 and miR-223-3p, respectively. The relative expression of the amplified target genes was determined using the 2^-△△ct^ method. Primers (Table 1) were designed and synthesized by JRDUN Biotechnology Co., Ltd (Shanghai, China).

**Table 1:** Primer Sequence.

Gene	Sequence(5’-3’)
miR-223-3p	Forward: GTGCAGGGTCCGAGGT Reverse: CGGGCTGTCAGTTTGTCA
U6	Forward: CTCGCTTCGGCAGCACA Reverse: AACGCTTCACGAATTTGCGT
NLRP3 mRNA	Forward: GCAGCAAACTGGAAAGGAAG Reverse: CTTCTCTGATGAGGCCCAAG
β-actin	Forward: AGGGGCCGGACTCGTCATACT Reverse: GGCGGCACCACCATGTACCCT

#### Immunoblotting assay

Total protein of the cell lysate (25 µg) was separated using electrophoresis followed by transferring to PVDF membrane and blocking with 5% milk. The samples were incubated with rabbit monoclonal primary antibodies against NLRP3 (1:5000), Bax (1:5000), Caspase-3 (1:5000), Bcl-2 (1:5000), and ß-actin (1:10,000) at 4 °C overnight followed by reacting with the secondary antibodies conjugated with HRP. Bands of the immunoblotting were visualized using chemiluminescence, densitometrically scanned using a gel image analysis system, and gray scale values were analyzed using the Image J software.

### Statistical analysis

All statistical analyses were performed using SPSS version 26.0 (USA). Normally distributed data were presented as means ± SD. Independent samples *t*-test and one-way ANOVA were used for comparison between two groups and among multiple groups, respectively. Where ANOVA indicated a significant interaction between variables, post hoc analysis was performed using the Bonferroni test. *P* < 0.05 was considered statistically significant.

## Results

### Effect of pae on HG-induced MCMEC survival and apoptosis

**Figure 1:**
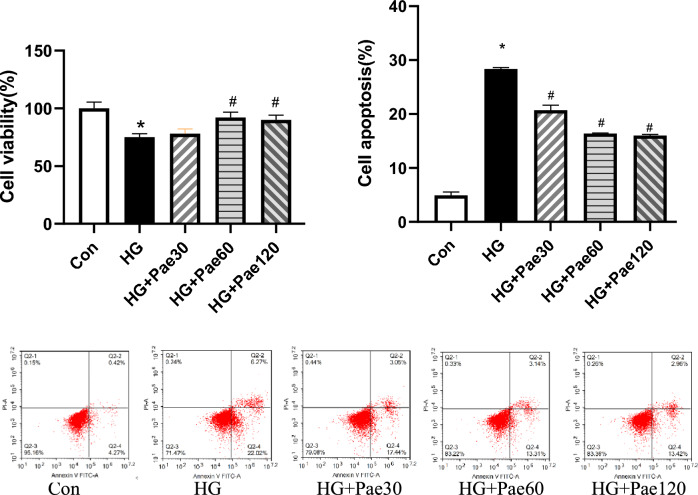
Effects of Pae on HG-induced MCMECs viability and apoptosis rate were tested using MTT assay followed by absorbance measurement with a microplate reader at 570 nm and flow cytometry, respectively. All data are expressed as mean ± SD. Con group (n = 3) and HG group were treated with 5.5 mmol/L glucose and 25 mmol/L glucose for 24 h, respectively. HG + Pae30, HG + Pae60 and HG + Pae120 groups were treated with 25 mmol/L glucose for 24 h followed by 30, 60 and 120 μmol/L Pae, respectively. *P < 0.01 vs. Con group. ^#^P < 0.05 vs. HG group.

To investigate the effect of Pae on the cell viability in the presence of high concentration of glucose, MCMECs were exposed to 25 mmol/L glucose for 24 h followed by adding various concentrations of Pae (30, 60 or 120 μmol/L) into the cell culture medium for additional 24 h. Cell viability and apoptosis were then analyzed using the MTT assay and flow cytometry, respectively. As shown in Fig. 1, HG significantly reduced cell viability but increased apoptosis of the cells. Low concentration of Pae (30 µmol/L) slightly but not significantly reversed reduction of the cell viability in the presence of HG, but it significantly blocked HG-induced apoptosis (*P* < 0.05, Fig. 1). At higher concentrations (60 and 120 µmol/L), Pae could not only significantly reversed the cell viability (*P* < 0.05, Fig. 1), but also significantly blocked apoptosis of the cells in response to HG (*P* < 0.05, Fig. 1). Since 60 μmol/L for 24 h treatment was demonstrated as the optimal condition for the study on MCMECs (Fig. 1), it was chosen in the subsequent experiments.

### Effect of pae on miR-223-3p and NLRP3 mRNA expression in MCMECs

**Figure 2:**
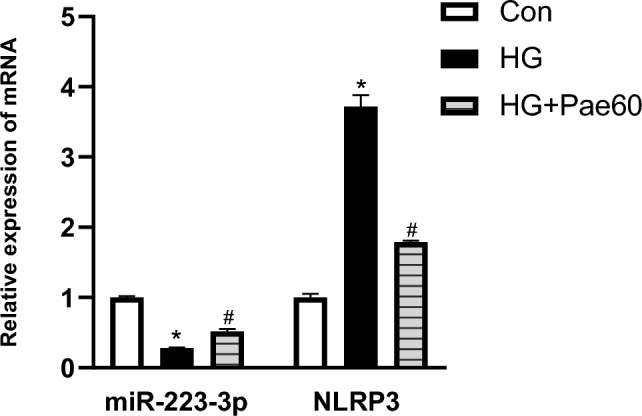
Effects of Pae on miR-223-3p expression and NLRP3 mRNA were analyzed using JRDUN PCR assay. MCMECs were pretreated with HG (25 mmol/L) for 24 h and stimulated with Pae (60 μmol/L) for another 24 h. Subsequently, miR-223-3p and NLRP3 mRNA levels were tested. Data are expressed as mean ± SD. n = 3. *P < 0.01 *vs.* Con group. ^#^*P* < 0.05 *vs.* HG group.

To test whether modulation of miR-223-3p by Pae in MCMECs attributed to the protective effect of Pae on cell survival, the expression of miR-223-3p in response to HG with or without subsequent Pae treatment was quantified using qRT-PCR. As shown in Fig. 2, compared to the control, miR-223-3p expression was significantly suppressed in the presence of HG, which was partially but significantly reversed by Pae treatment. In contrast, expression of NLRP3 mRNA was significantly upregulated in the presence of HG, which was also significantly reversed by the subsequent Pae treatment (*P* < 0.05, Fig. 2).

### Effect of pae on protein levels of NLRP3, Bax, Caspase-3, and Bcl-2 in the MCMECs in response to HG

**Figure 3:**
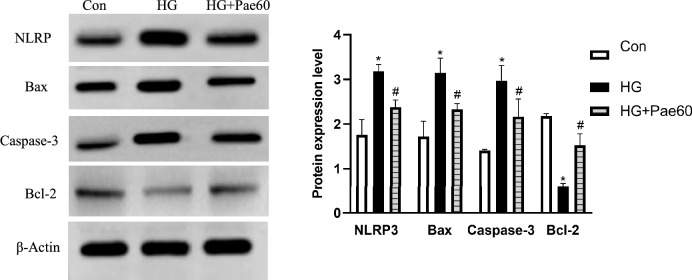
Effect of Pae on NLRP3 and apoptosis-associated proteins level based on Western blotting. MCMECs were pretreated with HG (25 mmol/L) for 24 h and stimulated with Pae (60 μmol/L) for another 24 h. Subsequently, NLRP3 and apoptosis-related proteins levels were measured using western blot assay. All data are expressed as mean ± SD. n = 3. *P < 0.05 *vs.* Con group. ^#^*P* < 0.05 *vs.* HG group.

Next, effect of Pae (60 μmol/L, for 24 h) on the apoptosis-associated proteins and inflammasome NLRP3 was semi-quantitively assessed by immunoblotting. As shown in Fig. 3, HG treatment resulted in significant upregulation of NLRP3, Bax and Caspase-3, but significant downregulation of Bcl-2 protein level (Fig. 3 and supplemental Fig. S1). Notably, Pae could partially but significantly reversed the effects of HG on synthesis of the apoptosis-associated proteins (Bax, Caspase-3, and Bcl-2) as well as inflammasome NLRP3 (*P* < 0.05, Fig. 3 and supplemental Fig. S1).

### Effect of exogenous miR-223-3p mimics and inhibitors on HG-induced MCMEC survival and apoptosis

**Figure 4:**
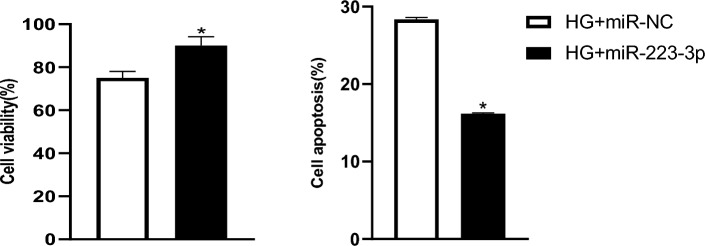
Effects of overexpression of miR-223-3p on HG-induced MCMECs viability and apoptosis rate. MCMECs were transfected with MiR-223-3p mimic or miR-NC and then treated with HG (25 mmol/L) for 24 h. Cell viability and apoptosis rate were tested using MTT assay followed by absorbance measurement using a microplate reader at 570 nm and flow cytometry, respectively. All data are expressed as mean ± SD. n = 3. *P < 0.05 *vs.* HG + miR-NC group.

To further explore the role of miR-223-3p in mediating HG-induced MCMEC apoptosis, the MCMECs were transfected with either exogenous miR-223-3p mimics or miR-NC followed by exposing to HG (25 mmol/L) for 24 h. As shown in Fig. 4, transfection of exogenous miR-223-3p mimics into the cells resulted in not only significantly increased cell survival, but also significant attenuation of apoptosis in the MCMECs (*P* < 0.05, Fig. 4).

### Effect of exogenous miR-223-3p mimics on the expression of NLRP3 and apoptosis-related proteins

To confirm the potential links between miR-223-3p and NLRP3, first, expression of NLRP3 and miR-223-3p was assessed in the MCMECs following transfection of exogenous miR-223-3p mimics. As shown in the supplemental Fig. S2, transfection of exogenous miR-223-3p mimics into the MCMECs resulted in significant suppression of NLRP3 expression in the control cells but not in the cells that overexpressing NLRP3 (Fig. S2A), and moreover, upregulation of miR-223-3p in the cells was not affected by the status of NLRP3 expression (Fig. S2B).

**Figure 5:**
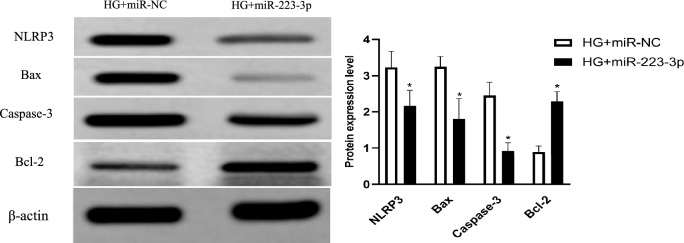
Effects of overexpression of miR-223-3p on NLRP3 and apoptosis-associated proteins level. MCMECs were transfected with MiR-223-3p mimic or miR-NC, pretreated with HG (25 mmol/L) for 24 h and stimulated with Pae (60 μmol/L) for another 24 h. NLRP3 and apoptosis-related proteins levels were measured using western blot assay. All data are expressed as mean ± SD. n = 3. *P < 0.05 *vs.* HG + miR-NC group.

Next, since suppression of NLRP3 by siRNA could not only significantly stimulate MCMEC proliferation (supplemental Fig. S3A), but also inhibition of apoptosis (supplemental Fig. S3B), protein levels of NLRP3 and apoptosis-associated proteins were semi-quantitively assessed by immunoblotting in the cells following transfection of miR-223-3p mimics. As shown in Fig. 5, overexpression of miR-223-3p resulted in significant reduction in the protein levels of NLRP3, Bax and Caspase-3, but significant increase of Bcl-2 protein in the presence of HG (*P* < 0.05, Fig. 5 and supplemental Fig. S4).

### Effect of exogenous miR-223-3p inhibitors plus pae on cell survival and apoptosis

**Figure 6:**
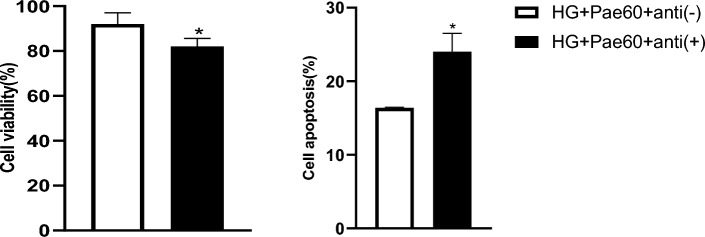
Effects of miR-223-3p knockdown combined with Pae on cell viability and apoptosis rate. MCMECs were transfected with anti-miR-223-3p [anti( +)] or anti-miR-NC [anti(−)], pretreated with HG (25 mmol/L) for 24 h and stimulated with Pae (60 μmol/L) for another 24 h. Cell viability and apoptosis rate were tested using MTT assay followed by absorbance measurement with a microplate reader at 570 nm and flow cytometry, respectively. All data ware expressed as mean ± SD. n = 3. *P < 0.05 *vs.* HG + Pae60 + anti( −) group.

To further explore the role of miR-223-3p in mediating the blockade of HG-induced MCMEC apoptosis by Pae, the cells were also transfected with exogenous miR-223-3p specific inhibitors [anti ( +)] or scramble anti-miR-NC [anti (−)] prior to be exposed to HG (25 mmol/L) for 24 h plus Pae (60 μmol/L) for additional 24 h. As shown in Fig. 6, viability of the cells transfected with specific miR-223-3p inhibitors [anti ( +)] was significantly lower than that of the cells transfected with scramble anti-miR-NC [anti (−)] (Fig. 6). In contrast, proportion of the apoptotic cells was significantly higher in the MCMECs transfected with specific miR-223-3p inhibitors [anti ( +)] compared to the cells transfected with scramble anti-miR-NC [anti (−)] (*P* < 0.05, Fig. 6) even in the presence of Pae.

### Effect of exogenous miR-223-3p inhibitors plus pae on the expression of NLRP3 and apoptosis-related proteins

**Figure 7:**
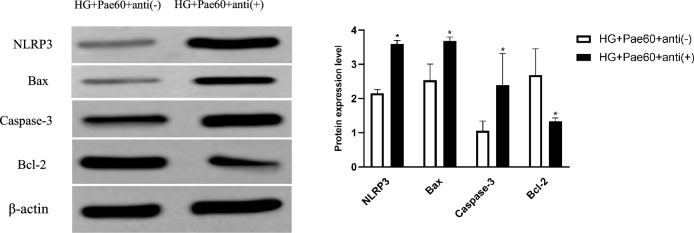
Effects of miR-223-3p knockdown combined with Pae on NLRP3 and apoptosis-associated proteins level. MCMECs were transfected with anti-miR-223-3p [anti( +)] or anti-miR-NC [anti(-)] pretreated with HG (25 mmol/L) for 24 h and stimulated with Pae (60 μmol/L) for another 24 h. Finally, NLRP3 and apoptosis-related proteins levels were measured using western blot assay. All data are expressed as mean ± SD. n = 3. *P < 0.05 *vs.* HG + Pae60 + anti( −) group.

Next, protein levels of NLRP3 and apoptosis-associated proteins were assessed in the MCMECs transfected with either exogenous specific miR-223-3p inhibitors [anti ( +)] or negative anti-miR-NC [anti (−)] followed by treatment with HG and Pae. As shown in Fig. 7, protein levels of NLRP3, Bax and Caspase-3 were significantly upregulated, while Bcl-2 was significantly downregulated, in the cells transfected with specific miR-223-3p inhibitors [anti ( +)] compared to the cells transfected with scramble anti-miR-NC [anti (−)] (*P* < 0.05, Fig. 7 and supplemental Fig. S5) even in the presence of Pae.

## Discussion

Vascular endothelial cell dysfunction is characterized not only by disturbances in vasoconstriction and relaxation, but also by enhanced endothelial inflammation, leukocyte adhesion, oxidative stress, increased endothelial proliferation and migration, and impaired endothelial barrier function^8,12,13^. Vascular endothelial cell dysfunction contributes to the development of vascular complications in diabetes^14,15^ and could therefore be an ideal target for treatment of these complications^16^.

Pae, a phenolic compound extracted from moutan bark, has been shown to have anti-inflammatory activities and to protect endothelial cells from apoptosis in response to variety kinds of insults including oxidized low-density lipoprotein (ox-LDL), lipopolysaccharide (LPS), and high glucose^8,10,17–19^. In this study, we demonstrated that Pae dramatically suppressed HG-induced apoptosis in the MCMECs, that Pae significantly stimulated gene expression and protein synthesis of miR-223-3p and anti-apoptotic protein (Bcl-2), but significantly inhibited the inflammasome (NLRP3), pro-apoptotic protein (Bax), and caspase-3 in the MCMECs in the presence of HG, suggesting Pae modulate HG-induced apoptosis in the MCMECs via regulating expression of miR-223-3p and its target protein NLRP3 as well as down-stream apoptosis-associated proteins.

Glucose, as a damage-associated molecular pattern, is the first upregulation signal of NLRP3 and pro-IL-1β^20^. HG stimulation, which occurs partially via NLRP3/IL-1β pathway, induces macrophage polarization to M1 phenotype, which is a potential pathogenesis mechanism for diabetic complications^21^. Studies by Wan et al. showed that down-regulation of NLRP3 could suppress intercellular adhesion molecule-1 (ICAM-1) and vascular cell adhesion molecule-1 (VCAM-1) and reduce AS risk^22^. In vitro experiments showed that HG enhanced the expression of NLRP3 inflammasome and the secretion of IL-1β in human umbilical vein endothelial cells (HUVECs)^22^. HG also induces the activation of Toll like receptor-4, resulting in elevated nuclear factor-kappa B (NF-kB), which further activates the NLRP3 inflammasome, leading to increased proinflammatory cytokine expression^23^. Consistent with these results, in the current study, we demonstrated that HG induced the upregulation of NLRP3 as well as apoptosis of MCMECs and that suppression of NLRP3 by siRNA resulted in stimulation of cell proliferation as well as inhibition of apoptosis, suggesting NLRP3 play an important role in mediating apoptosis of MCMECs in response to HG.

MicroRNAs (miR) are a group of non-coding RNAs 18–24 nucleotides in length^24^, which can directly bind to the 3'-terminal untranslated region of specific mRNA target molecules to induce their degradation and inhibit protein synthesis. By which mechanism, miRNAs exert immune regulatory effects through a negative feedback regulation mechanism and participate in the development of tissue organ or cell injury^25^. In addition, Queries using the online software TargetScan revealed that miR-223-3p binds complementarily to the nucleotide sequence in the 3' part of NLRP3 mRNA^9,16^, indicating NLRP3 could be targeted by miR-223-3p. Consistent with these reports, the current study demonstrated that exogenous miR-223-3p mimic significantly suppressed NLRP3 as well as pro-apoptotic protein (Bax) and caspase-3, while significantly stimulated anti-apoptotic protein (Bcl-2), and that suppression of miR-223-3p by exogenous inhibitor resulted in significant upregulation of NLRP3, Bax, and Caspase-3, but downregulation of Bcl-2 in the MCMECs treated with HG, suggesting miR-223-3p could play an important role in modulating mouse vascular endothelial cell survival or apoptosis in response to HG via a mechanism of regulating NRLP3 as well as apoptosis-associated proteins.

Previous studies have reported that Pae attenuated inflammatory response of human umbilical vein endothelial cells (HUVECs) via stimulating monocytes (THP-1)-derived exosomal miR-223^26^. It has also been reported that Pae inhibited apoptosis, oxidative stress, inflammatory response in HUVECs in response to HG stimulation via regulating SIRT1/FOX3a/NF-kB pathway^10^, and that Pae inhibited apoptosis of HUVECs induced by oxidized low-density lipoprotein (ox-LDL)^11^. Moreover, our previous study had demonstrated that miR-223-3p reduced HG and high fat-induced endothelial cell injury in diabetic mice by regulating NLRP3 expression^9^. Consistent with these previous studies, in the current study, we further demonstrated that Pae significantly blocked HG-induced apoptosis of MCMECs, and that Pae significantly reversed downregulation of miR-223-3p by HG in the MCMECs. We further demonstrated that Pae significantly suppressed NLRP3 as well as pro-apoptotic protein (Bax) and caspase-3, but significantly stimulated anti-apoptotic protein Bcl-2 in the MCMECs in the presence of HG, suggesting Pae could rescue mouse vascular endothelial cells from HG-induced apoptosis via a mechanism of regulating miR-223-3p and its specific target NRLP3.

In conclusion, the current study demonstrated that Pae significantly blocked HG-induced apoptosis of MCMECs and the suppressive effect of HG on miR-223-3p expression in the MCMECs. In addition, proteins of inflammasome NLRP3 as well as apoptosis-associated proteins Bax, Bcl-2, and caspase-3 were significantly modulated by Pae in the MCMECs exposed to HG. These findings suggested that Pae attenuate HG-induced apoptosis of MCMECs via regulating miR-223-3p and its target protein of NLRP3.

### Supplementary Information

Supplementary Figure S1.

Supplementary Figure S2.

Supplementary Figure S3.

Supplementary Figure S4.

Supplementary Figure S5.

## Data Availability

All relevant data are within the paper.
