# Supplementary figures and images for: Paeonol attenuated high glucose-induced apoptosis via up-regulating miR-223-3p in mouse cardiac microvascular endothelial cells
Source: Sci Rep. 2024 Jul 19;14:16699. doi: 10.1038/s41598-024-67721-3 (PMC11271548; doi:10.1038/s41598-024-67721-3)

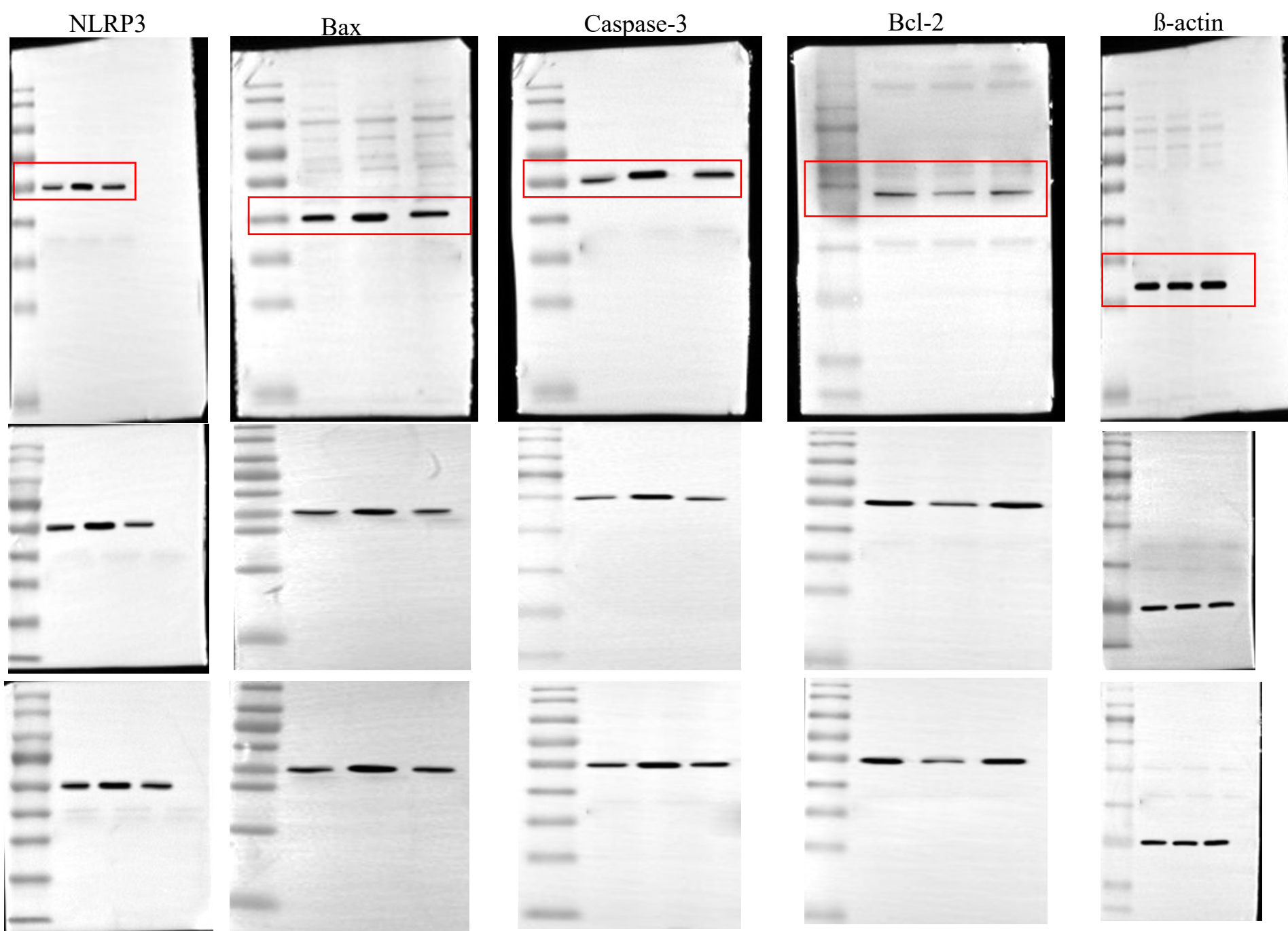

**Fig S1.** Images of original blotting for Figure 3.

Supplement: Supplementary file 1 — Supplementary Figure S1. [file 41598_2024_67721_MOESM1_ESM.pdf]

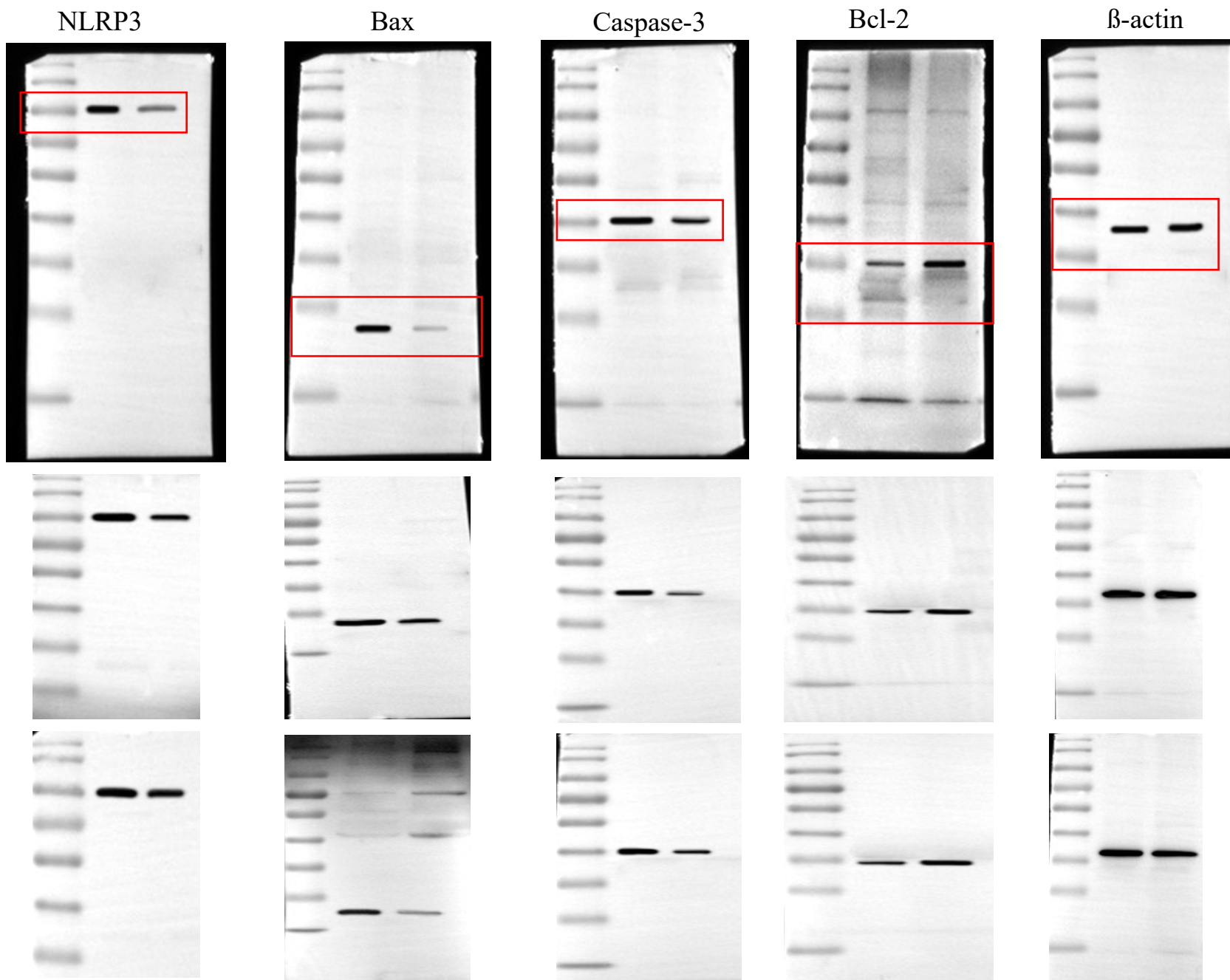

**Fig S4.** Images of original blotting for Figure 5.

Supplement: Supplementary file 4 — Supplementary Figure S4. [file 41598_2024_67721_MOESM4_ESM.pdf]

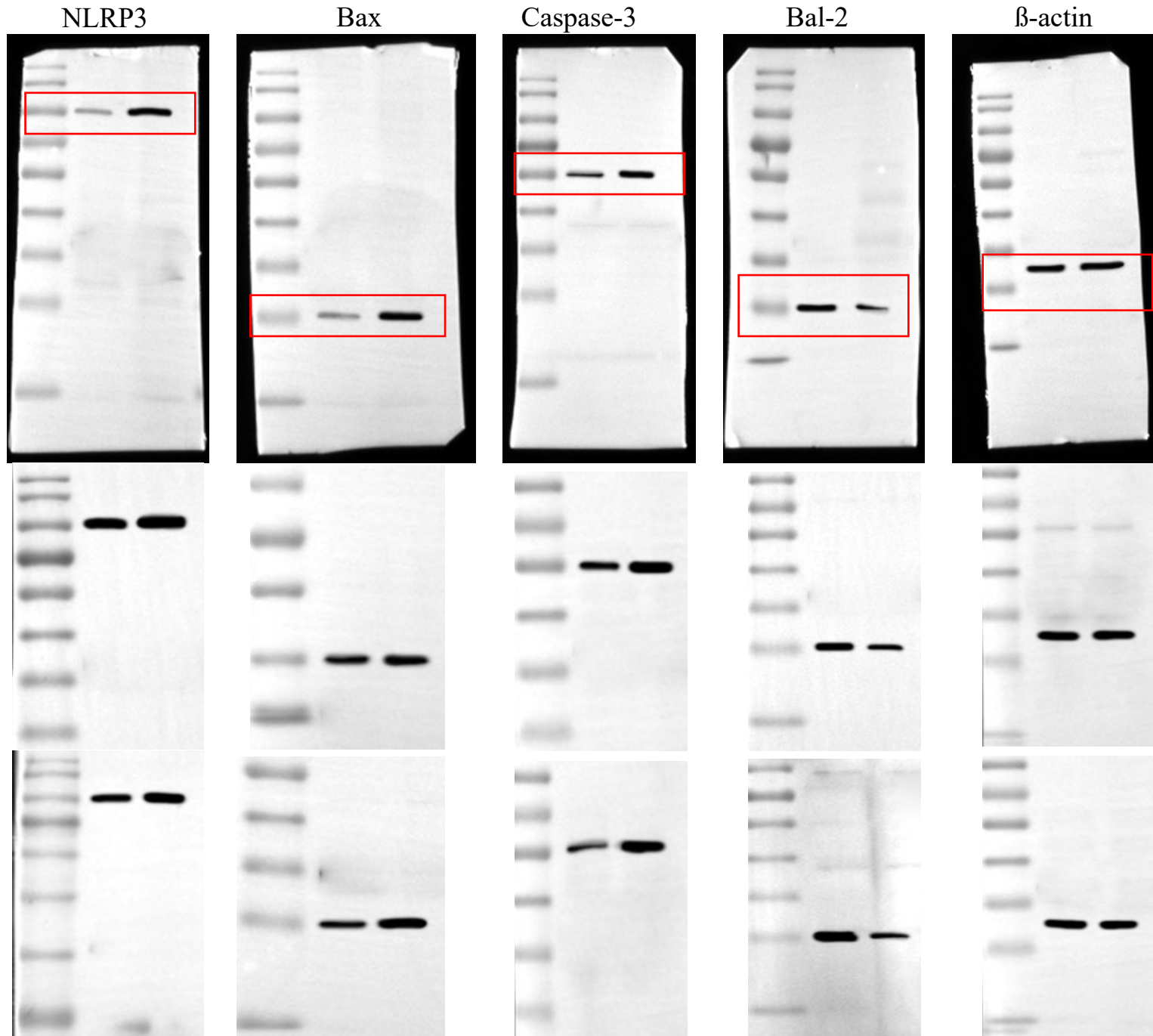

**Fig S5.** Images of original blotting for Figure 7.

Supplement: Supplementary file 5 — Supplementary Figure S5. [file 41598_2024_67721_MOESM5_ESM.pdf]
