# Supplementary material for: Paeonol attenuated high glucose-induced apoptosis via up-regulating miR-223-3p in mouse cardiac microvascular endothelial cells
Source: Sci Rep. 2024 Jul 19;14:16699. doi: 10.1038/s41598-024-67721-3 (PMC11271548; doi:10.1038/s41598-024-67721-3)
Supplement: Supplementary file 2 — Supplementary Figure S2. [file 41598_2024_67721_MOESM2_ESM.pdf]

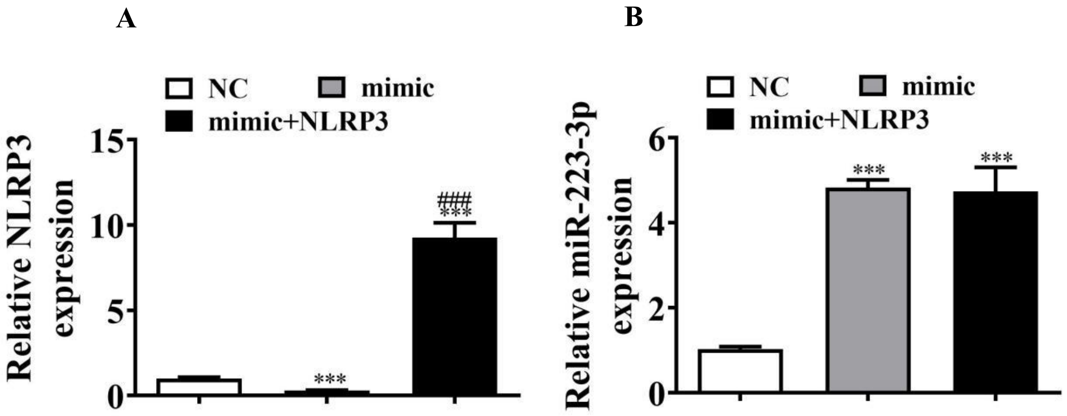


**Fig S2.** Effect of exogenous miR-223-3p mimics on the expression of NLRP3 and miR-223-3p. Negative control or miR-223-3p mimics were transfected into the MCMECs that overexpressing NLRP3. Relative expression of NLRP3 and miR-223-3p was then assessed by real time qRT-PCR as described in the methods. **Panel A:** Effect on NLRP3 expression. Vertical axis: relative expression of NLRP3; horizontal axis: cells treated as indicated. *** $P< 0.001$ compared to negative control (NC); ### $P< 0.001$ compared to the cells transfected with miR-223-3p mimics only (mimic). **Panel B:** Effect on miR-223-3p expression. Vertical axis: relative expression of miR-223-3p; horizontal axis: cells treated as indicated. *** $P < 0.001$ compared to negative control (NC).
