# Supplementary material for: Paeonol attenuated high glucose-induced apoptosis via up-regulating miR-223-3p in mouse cardiac microvascular endothelial cells
Source: Sci Rep. 2024 Jul 19;14:16699. doi: 10.1038/s41598-024-67721-3 (PMC11271548; doi:10.1038/s41598-024-67721-3)
Supplement: Supplementary file 3 — Supplementary Figure S3. [file 41598_2024_67721_MOESM3_ESM.pdf]

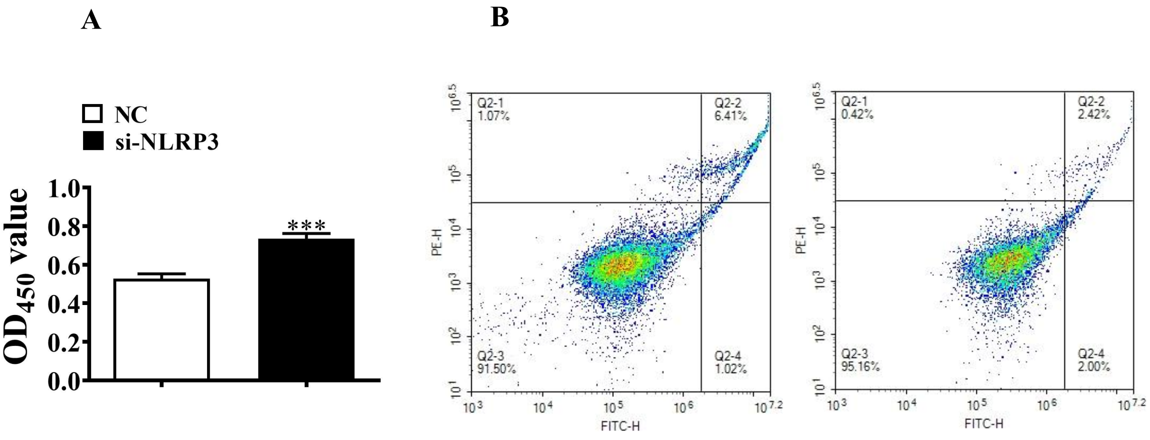

Fig S3. Effect of NLRP3 suppression by siRNA on cell proliferation and apoptosis. MCMECs were transfected with negative control (NC) or specific siRNA targeting NLRP3. Cell proliferation was then assessed using commercially available kit (CCK8) and apoptosis was assessed by flow cytometry. **Panel A:** Effect on cell proliferation. Vertical axis: OD value at 450nm; horizontal axis: cells transfected with negative or NLRP3 specific siRNA. *** $P< 0.001$ compared to negative control (NC). **Panel B:** Effect on apoptosis assessed by flow cytometry.
